# Supplementary material for: Fatty acid metabolism changes in association with neurobehavioral deficits in animal models of fetal alcohol spectrum disorders
Source: Commun Biol. 2023 Jul 17;6:736. doi: 10.1038/s42003-023-05127-z (PMC10352263; doi:10.1038/s42003-023-05127-z)
Supplement: Supplementary file 2 — Supplementary Information [file 42003_2023_5127_MOESM2_ESM.pdf]

Supplementary Figure Legends

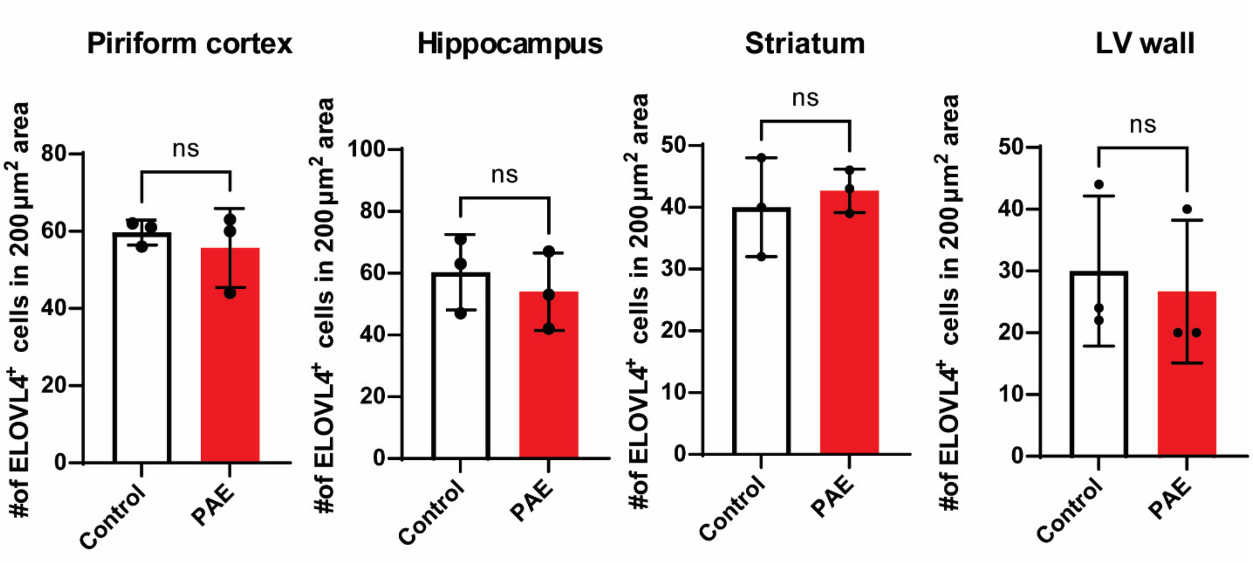

Supplementary Figure 1. The number of ELOVL4 expressing cells is not altered in the piriform cortex, striatum, lateral ventricular wall and hippocampus. There is no significant difference in the number of ELOVL4-positive cells in the indicated brain regions between PAE and control animals by Student's t-test. Control n=3, PAE n=3. Graphs represent mean ± SEM. Each dot represents an individual animal. ns = not significant.

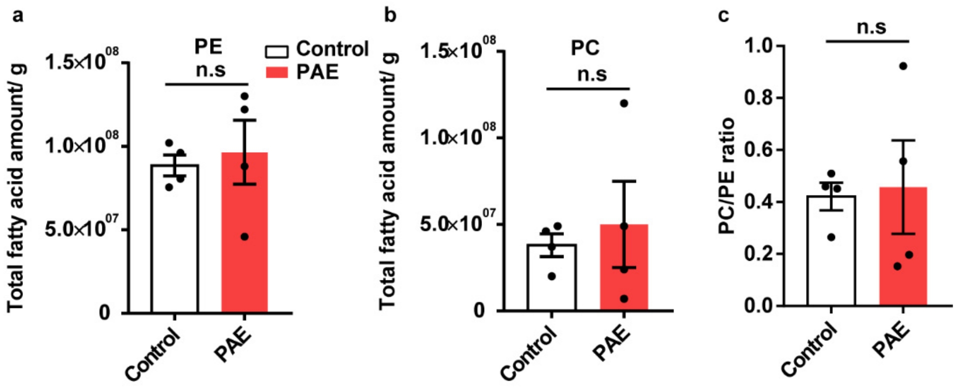

Supplementary Figure 2. PAE does not alter the total amount of PC or PE from the motor cortex at P30

(a-c) There is no significant difference in the total amount of PC (a) and PE (b) or the ratio of PC/PE (c) between PAE and control animals by Student's t-test. Control n=4, PAE n=4. Graphs represent mean ± SEM. Each dot represents an individual animal. PE = phosphatidylethanolamine, PC = phosphatidylcholine, n.s = not significant.

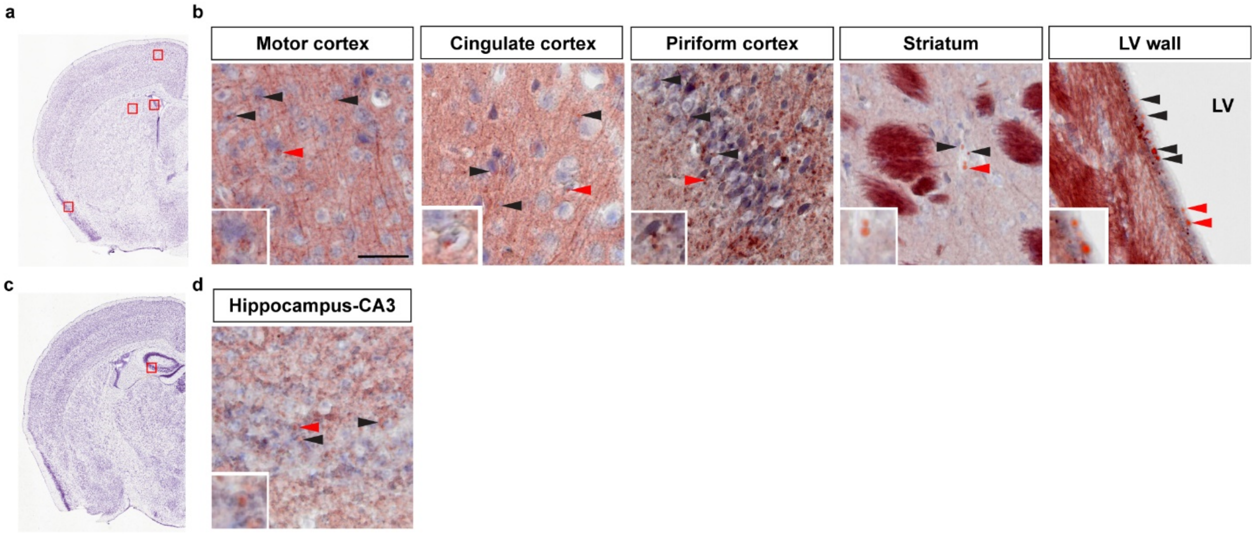

Supplementary Figure 3. Validation of the ORO staining using aging mouse brain

A 12 month old mouse brain was used to test ORO staining. (a, c) Red boxes in Allen Mouse Brain Atlas images show the brain areas where representative images were taken. (b, d) Arrowheads indicate LDs stained with ORO. Higher magnification views of the areas indicated by red arrowheads were presented in insets. Scale bar = 50µm. LV = lateral ventricle.

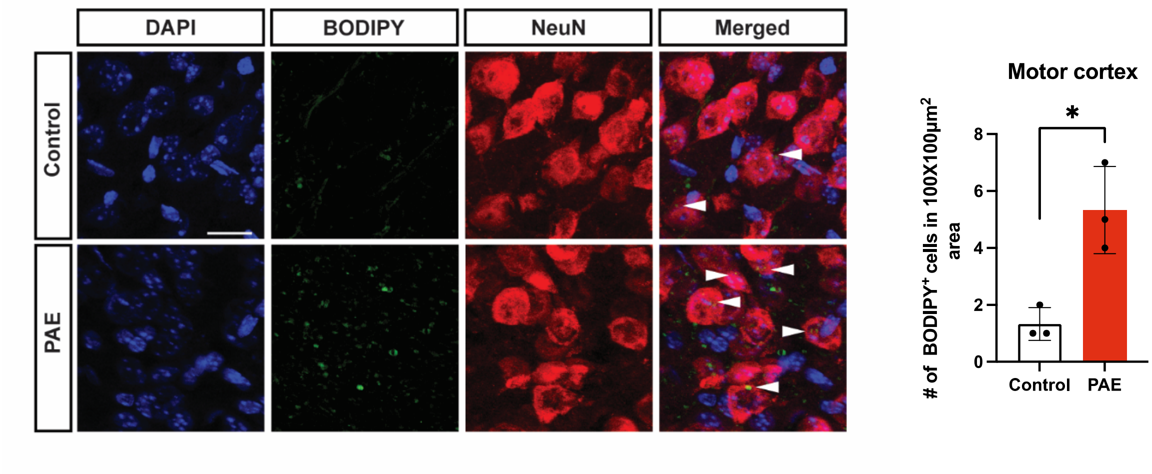

Supplementary Figure 4. BODIPY staining shows LD accumulation in neurons in the motor cortex of PAE mice at P30

Representative images of P30 control and PAE motor cortices stained with BODIPY. Arrowheads indicate LDs stained with BODIPY in NeuN positive cells. Scale bar = 20μm. Graph shows that the number of BODIPY-positive cells is significantly higher in the motor cortex of PAE mice compared with control mice at P30 ($p=0.02$). *$p<0.05$. Student's t-test. Control n=3, PAE n=3. Graphs represent mean ± SEM.

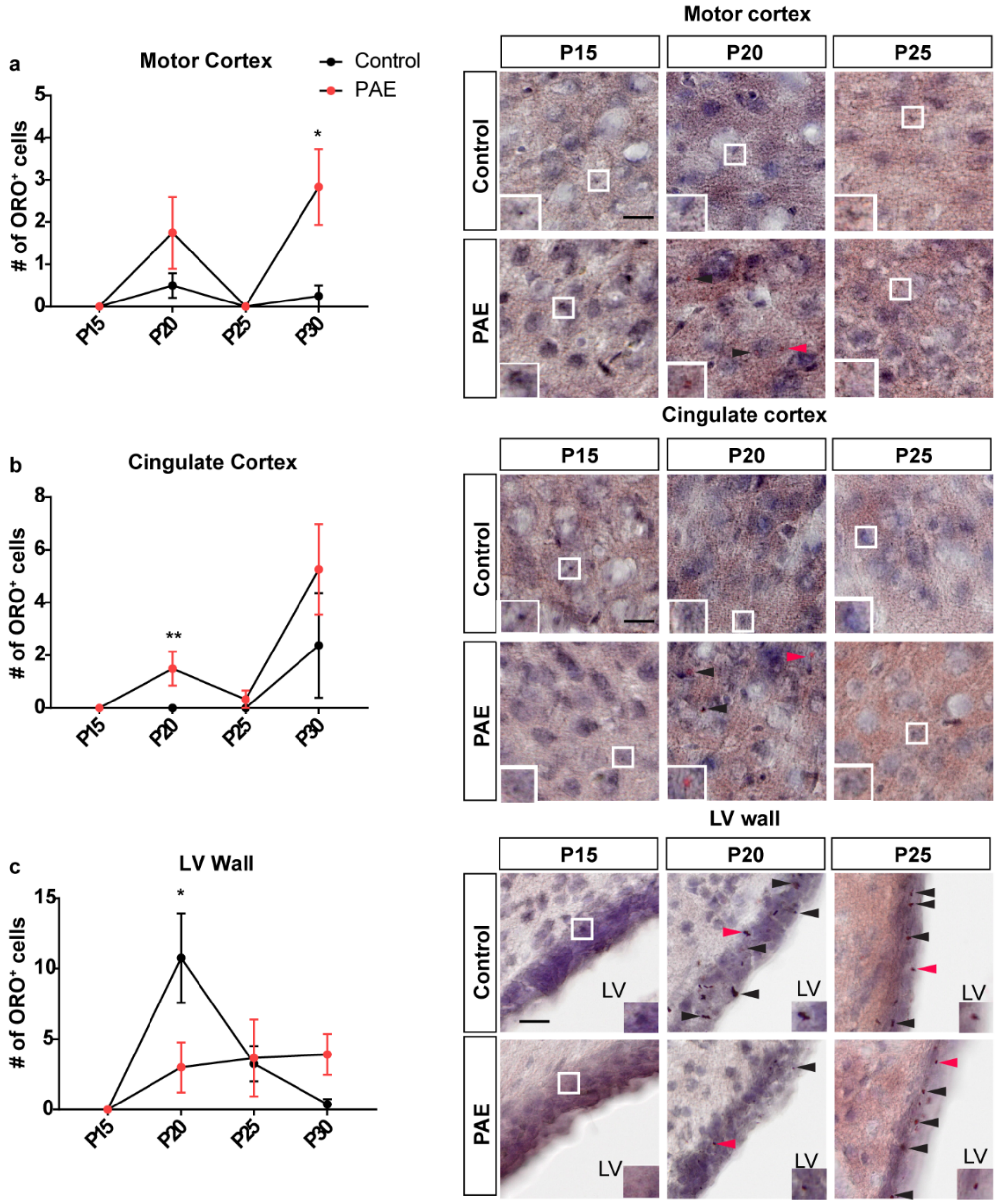

Supplementary Figure 5. LD accumulation shows dynamic change during postnatal brain development

(a-c) The number of LD accumulating cells is quantified in 200 x 200 μm^2 area for each brain region and compared between control and PAE mice. At P20, a slight increase of LD accumulating cells in the motor cortex of PAE is observed (p=0.1576). In addition, a significant increase of LD accumulating cells in the cingulate cortex of PAE mice (p=0.0056), whereas a significant increase of LD including cells in the LV wall of control mice (p=0.0226) is observed at P20. At P15 and P25, there is no difference between control

and PAE mice in all three brain regions. Two-way ANOVA showed no interaction between the prenatal exposure type and different time points of the observation. Bonferroni's post hoc test was used to detect statistical significance between control and PAE for each time point. n=4 per group for P15 and P20 time points. n=4 control and n=3 PAE for P25 time point. *$p<0.05$. Line graphs represent mean ± SEM. P30 data from Figure 3 is added to the graphs for reference. (d) Representative images of ORO staining. Arrowheads indicate LDs. Insets show higher magnification views around red arrowheads or white square of each panel. Scale bar = 20μm. LV = lateral ventricle.

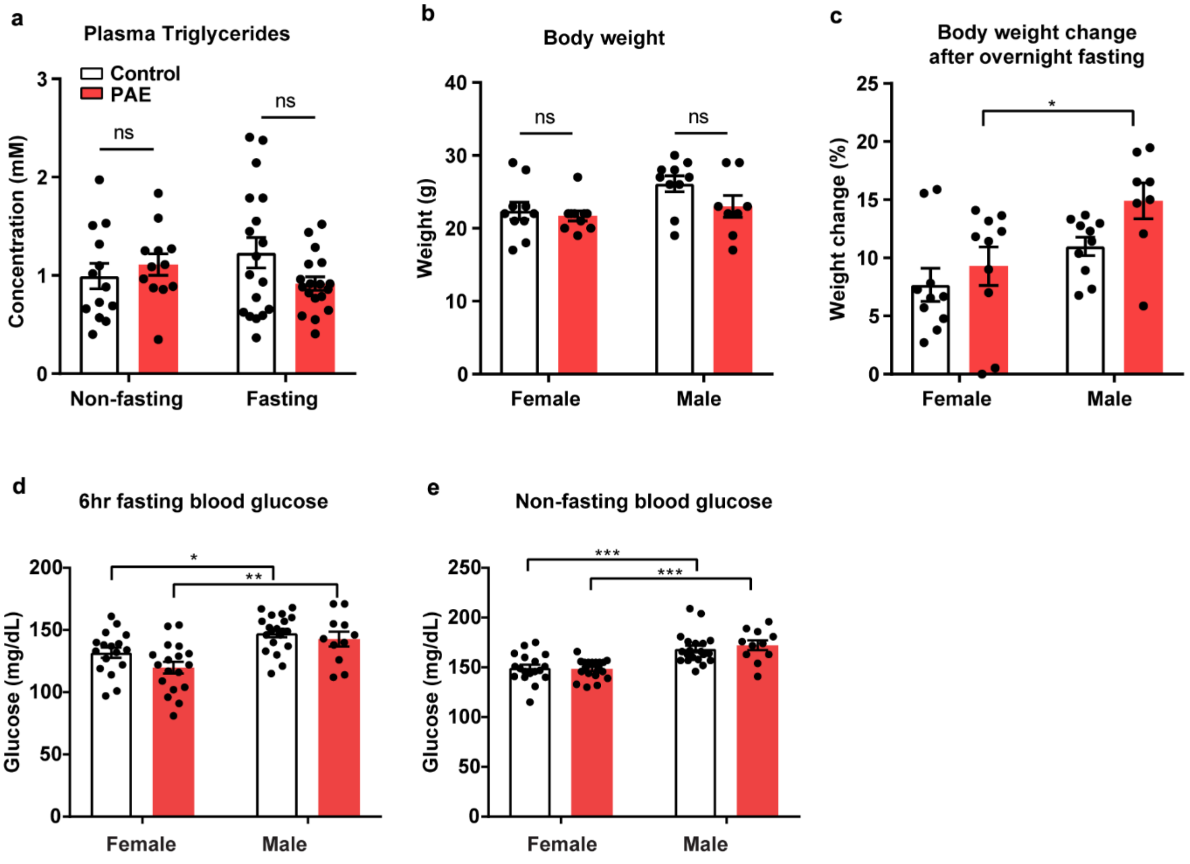

Supplementary Figure 6. PAE does not affect blood triglyceride, glucose levels, or body weight.

(a) No statistical significance is found between control and PAE for plasma triglycerides levels in non-fasted and overnight fasted (16hr) conditions. Although all data from both male and female are used for analysis, no sex dimorphism was observed in either control or PAE by Student's t-test. Fasting control n=18, Fasting PAE n=18, Non-fasting control n=13, Non-fasting PAE n=12. (b, c) There is no significant difference in body weights between control and PAE mice for both sexes. Body weight after overnight fasting was similar between control and PAE mice. However, in PAE group, male mice lose significantly more weight compared to the female mice after overnight fasting (p=0.0427). No interaction between sex and prenatal exposure type was found by two-way ANOVA. Tukey's multiple comparisons were used for the post hoc test. Control male = 10, Control female = 10, PAE male = 8, PAE female = 10. (d, e) Two-way ANOVA revealed no interaction between sex and prenatal exposure type. Tukey's multiple

comparison test showed a significant difference between the sexes. There is no significant difference in blood glucose levels between control and PAE mice in both non-fasting and 6 hours fasting conditions. 6 hr fasting: Control female vs Control male ($p=0.047$), and PAE female vs PAE male ($p=0.0074$). Non-fasting: Control female vs Control male ($p=0.0005$), PAE female vs PAE male ($p=0.0002$). 6hr fasting blood glucose: Control male n=20, Control female n=17, PAE male n=11, PAE female n=18; Non-fasting blood glucose: Control male=20, Control female n=18, PAE male n=11, PAE female n=19. All graphs represent mean ± SEM. Each dot represents an individual animal. *, **, *** $p<0.05$, 0.01, 0.001.

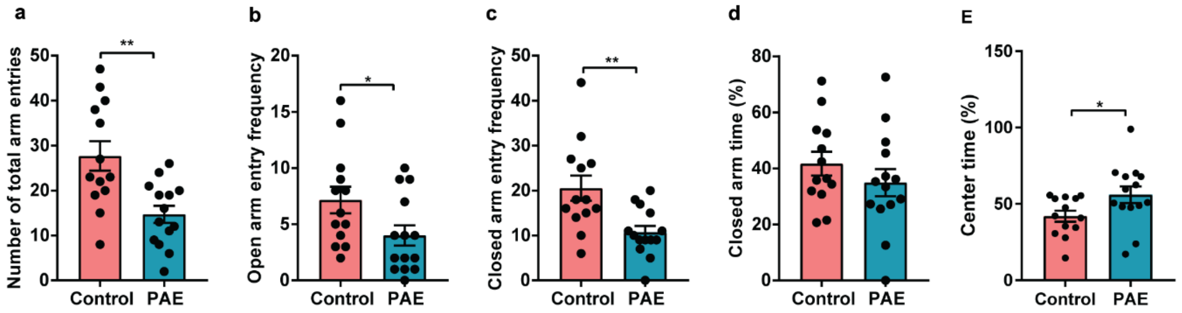

Supplementary Figure 7. Both numbers of arm entries and center time in EPM are significantly changed in PAE mice compared to control mice

(a) In EPM, the total number of arm entries (open and closed) is significantly decreased in PAE mice compared to control mice ($p=0.0018$). (b, c) The open arm and closed arm entry frequencies are significantly lower in PAE mice compared to control mice ($p=0.0433$ and $p=0.0038$, respectively). (d) No difference in closed arm time is observed between PAE and control mice. PAE mice spend significantly more time in the center of EPM compared to control mice ($p=0.0440$). Control n=13, PAE n=14. *, ** $p<0.05$, 0.01. Student's t-test. All graphs represent mean ± SEM. Each dot represents an individual animal.

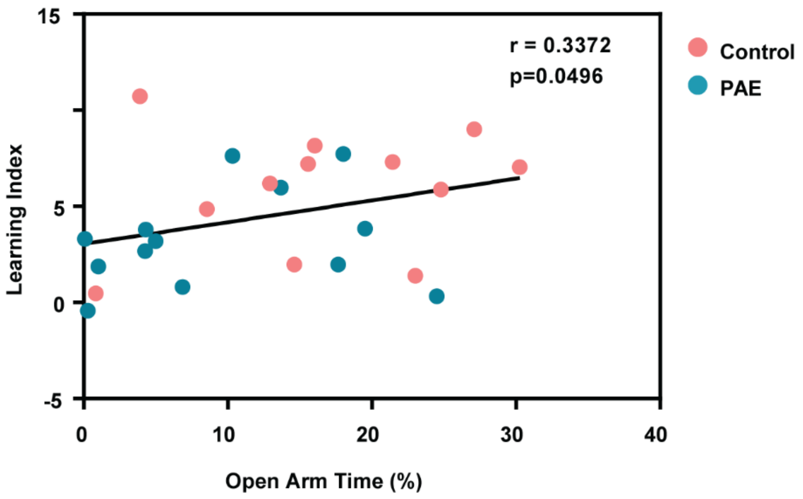

Supplementary Figure 8. Anxiety phenotype is correlated with poor motor learning behavior.

There is a positive correlation between the learning index and open arm time (%) by Pearson's correlation analysis. Control n=12, PAE n=13. Each dot represents an individual animal.

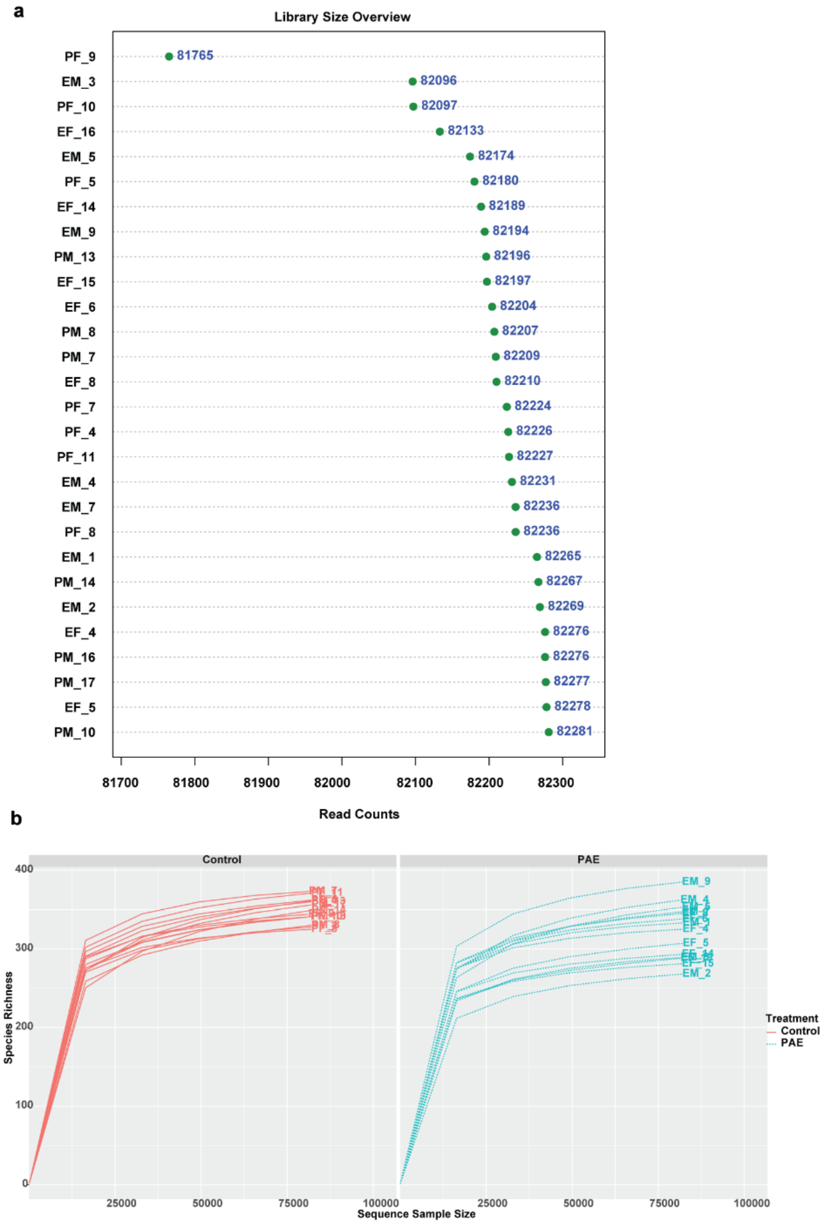

Supplementary Figure 9. Quality control of 16s rRNA sequencing.

(a) Library sizes of samples range from 81765 to 82281 reads. (b) Rarefaction curves show species richness and diversity. Each line represents a sample that is derived from an individual mouse. PF = Control (PBS exposed) female, PM = Control male, EF = PAE female, EM = PAE male.

Level 2 pathway classified by Tax4fun2

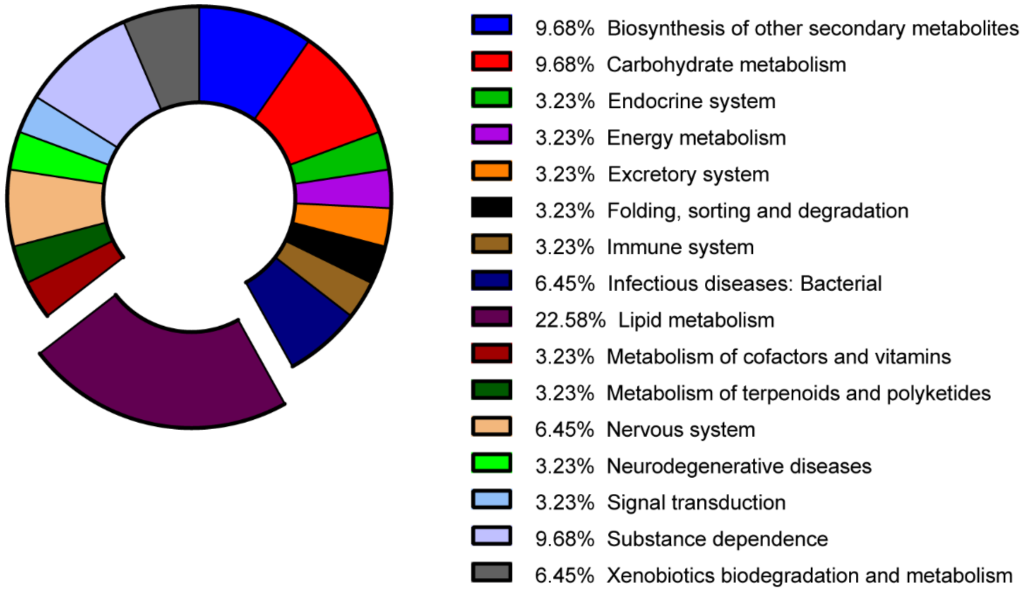

Supplementary Figure 10. Functional profiles altered by PAE in the microbial community.

Pie chart presents the percentages of significantly altered ($p<0.05$ by Welch's t-test) pathways in PAE mice compared to the control. Actual percentages of each pathway are indicated in the legend. Lipid metabolism is the profile that shows the largest difference between microbial communities of PAE and control mice.
